# Overexpression of miR-29 Leads to Myopathy that Resemble Pathology of Ullrich Congenital Muscular Dystrophy

**DOI:** 10.3390/cells8050459

**Published:** 2019-05-15

**Authors:** Chuncheng Liu, Lei Li, Mengxu Ge, Lijie Gu, Meng Wang, Kuo Zhang, Yang Su, Yuying Zhang, Chang Liu, Miaomiao Lan, Yingying Yu, Tongtong Wang, Qiuyan Li, Yaofeng Zhao, Zhengquan Yu, Ning Li, Qingyong Meng

**Affiliations:** 1 Beijing Advanced Innovation Center for Food Nutrition and Human Health, College of Biological Science, China Agricultural University, Beijing 100193, China; liuchuncheng.china@gmail.com (C.L.); lilei1995@cau.edu.cn (L.L.); mengxuge@gmail.com (M.G.); gulj@cau.edu.cn (L.G.); mengwang@cau.edu.cn (M.W.); zhangkuo@cau.edu.cn (K.Z.); suyangsy@aliyun.com (Y.S.); yuying0609@126.com (Y.Z.); ChangL@cau.edu.cn (C.L.); miaomiaolan@cau.edu.cn (M.L.); yuyingyingqf3y@163.com (Y.Y.); vetwangtongtong@163.com (T.W.); liqiuyan@cau.edu.cn (Q.L.); yaofengzhao@cau.edu.cn (Y.Z.); zyu@cau.edu.cn (Z.Y.); ninglcau@cau.edu.cn (N.L.); 2 The State Key Laboratory for Agrobiotechnology, College of Biological Sciences, China Agricultural University, Beijing 100193, China; liqiuyan@cau.edu.cn (Q.L.); yaofengzhao@cau.edu.cn (Y.Z.); ninglcau@cau.edu.cn (N.L.); 3 The Institute of Bioengineering and Technology, Inner Mongolia University of Science and Technology, Baotou 014010, China

**Keywords:** UCMD, disease model, miR-29, dysplasia, collagen

## Abstract

Ullrich congenital muscular dystrophy (UCMD) bring heavy burden to patients’ families and society. Because the incidence of this disease is very low, studies in patients are extremely limited. Animal models of this disease are indispensable. UCMD belongs to extracellular matrix-related diseases. However, the disease models constructed by knocking out some pathogenic genes of human, such as the *Col6a1*, *Col6a2*, or *Col6a3* gene, of mice could not mimic UCMD. The purpose of this study is to construct a mouse model which can resemble the pathology of UCMD. miR-29 is closely related to extracellular matrix deposition of tissues and organs. To address this issue, we developed a mouse model for overexpression miR-29 using Tet-on system. In the muscle-specific miR-29ab1 cluster transgenic mice model, we found that mice exhibited dyskinesia, dyspnea, and spinal anomaly. The skeletal muscle was damaged and regenerated. At the same time, we clarify the molecular mechanism of the role of miR-29 in this process. Different from human, *Col4a1* and *Col4a2*, target genes of miR-29, are the key pathogenic genes associating with these phenotypes. This mouse model simulates the human clinical and pathological characteristics of UCMD patients and is helpful for the subsequent research and treatment of UCMD.

## 1. Introduction

Ullrich congenital muscular dystrophy (UCMD) belongs to collagen VI-related myopathy. Collagen VI-related myopathies are caused by mutations of *COL6A1*, *COL6A2*, and *COL6A3*, which encode the collagen VI alpha chain [1]. *COL6A1* and *COL6A2* lie on chromosome 21, and *COL6A3* lies on chromosome 2. The dominant and recessive mutations of *COL6A1*, *COL6A2*, and *COL6A3* constitute a series of muscle diseases, ranging from mild terminal Bethlem myopathy (BM) to severe UCMD and a series of intermediate phenotypes between the two extremes [2,3].

Although UCMD is less prevalent than Duchenne muscular dystrophy, muscle damage is more serious in patients with UCMD. Before birth, UCMD patients presented less fetal movement. After birth, UCMD patients showed dystonia; joint abnormality; excessive flexibility of fingers, wrists, ankles, and so on [1].

UCMD patients can learn to turn over, crawl, and maintain a certain sitting posture during their growth; however, patients who are 5 to 15 years old usually lose walking ability. In patients with UCMD, spinal column fixation is required for scoliosis. The weakening of the diaphragm and other respiratory muscles causes respiratory failure in patients, and the patient needs assisted breathing. Respiratory-associated skeletal muscle failure is the main cause of death in adolescents [4,5,6,7].

For rare diseases, patient studies are limited, and the inherent variability of symptoms often leads to ambiguous results. Animal models of diseases are important for the research and treatment of these diseases.

A genetic mouse disease model of *Col6a1* deficiency was generated by knockout the *Col6a1*, and the homozygous mutants lost collagen VI in the skeletal muscle. However, the extracellular matrix of this mouse model is intact, which is only suitable as a mouse model of Bethlem myopathy [8].

At the same time, detection of *Col6a2* knockout mice by high-throughput platform showed that the grip strength of the mice was reduced and no other abnormalities were found [9,10]. Researchers also constructed a mouse model by gene targeting to induce abnormal splicing of *Col6a3* mRNA [11] and a mouse model knocked out the exon 16 of the *Col6a3* gene [12]. However, neither of these mice could mimic UCMD.

So how can we build a suitable mouse model that can recapitulate features of UCMD? Excessive accumulation of extracellular matrix proteins can cause fibrosis of tissues and organs, and the absence of extracellular matrix proteins can lead to collagen VI-related myopathy or skeletal muscle diseases related to the integrity of cell membranes and extracellular matrix. miR-29 can directly inhibit the expression of more than 20 kinds of extracellular matrix-related genes, including collagen, elastin, and integrin proteins, which are important components of extracellular matrix [13,14].

To address these issues, we developed a mouse model for overexpression miR-29 using Tet-on system. In the muscle-specific miR-29ab1 cluster dual transgenic (dTG) mice model, we found that mice exhibited dyskinesia, the skeletal muscle was necrotic with an abnormal extracellular matrix (ECM) structure. The partially dTG mice displayed respiratory disturbances or severe kyphos. Mechanism analysis reveal that the absence of *Col4a1* and *Col4a2*, the component of ECM and target genes of miR-29, cause the phenotypes, just like the performance of collagen VI mutations in human. These muscle-specific miR-29ab1 cluster dTG mice constitute an excellent abnormal skeletal muscle basement membrane model that closely resemble pathology of UCMD.

## 2. Materials and Methods

### 2.1. Ethics Statement

This study was approved by the Institutional Animal Care and Use Committee of China Agricultural University (SKLAB-2015-01-03).

### 2.2. Mice

To generate TRE-miR-29 mice, the mmu-miR-29a and mmu-miR-29b coding sequences were amplified by PCR from mouse genomic DNA (Forward Primer: 5′-GGACTTCACCTTCCCTCTCC-3′ and Reverse primer: 5′-ATTGGTTTGGCCCTTTATCC-3′), cloned into pTRE2 plasmid (Clontech) to generate pTRE-miR-29a,b1 cluster construct.

To generate ACTA1-rtTA mice, the promoter sequences were amplified by PCR from mouse genomic DNA (Forward Primer: 5′-TACTTGCCAGAGGTGACGGA-3′ and Reverse primer: 5′-GCTCTGACTCTGGCCCTGGG-3′), cloned into pTet-on plasmid (Clontech) instead of PCMV promoter to generate ACTA1-rtTA construct [15].

The TRE-miR-29a,b1 cluster transgenic mice and ACTA1- rtTA transgenic mice were generated using KUNMING mice, and backcrossed into the C57BL/6 mouse strain.

Generation of dual transgenic mice by crossing the TRE-miR-29a,b1 cluster mice and ACTA1-rtTA mice.

Mice were raised in controlled temperature (25 ± 1 °C) and humidity (60–70%) with a 12 h light, 12 h dark cycle.

The Evans Blue Dye (EBD) was injected i.p. into 28-day-old dTG mice and control mice 1 day before the sample collection [16].

### 2.3. Micro-Computed Tomography Experiment

The mice were anesthetized with sodium pentobarbital through i.p. then transferred to the in vivo Micro-computed tomography (Micro-CT) imaging system (Quantum FX, Caliper) [17]. Images were obtained with imaging software SimpleViewer.

### 2.4. Exercise

First, we observed the ability of the mouse to walk and turn over. When the dTG mice walked and turned over “normally”, each mouse used a mouse swimming pool for 10 min of exercise. When the “normal” dTG mice consumed physical energy by swimming, they were tested on their ability to walk and turn over. The pool was filled with distilled water and the temperature of the water was maintained at 30 °C. During the time the mice were swimming, attention was paid to the prevention of drowning in mice.

### 2.5. Histology, Immunochemistry, and Immunofluorescence

Skeletal muscles were cut, fixed in 4% PFA overnight, and then embedded in common paraffin. The antigen retrieval and 5% goat serum blocking were needed before the addition of the primary antibody. Immunostaining was performed with anti-Pax7 (Developmental Studies Hybridoma Bank, DSHB), anti-eMyHC (Developmental Studies Hybridoma Bank, DSHB), anti-Laminin (L9393, sigma), and anti-Collagen IV (ab6586, abcam) overnight at 4 °C [18]. After washing with PBS, the sections were incubated with Alexa 488-labeled goat anti-mouse Immunoglobulin G (Invitrogen, A-11034)/Alexa 594-labeled goat anti-mouse Immunoglobulin G (Invitrogen, A-11032)/Alexa 488-labeled goat anti-Rabbit Immunoglobulin G (Invitrogen, A-11008)/Alexa 594-labeled goat anti-Rabbit Immunoglobulin G (Invitrogen, A-11037) 1 h at room temperature.

### 2.6. Cell Culture

C2C12, mouse skeletal myoblasts were purchased from Beijing’s Cell Resource Center. C2C12 cells were cultured with DMEM, 10% FBS and 1% penicillin and streptomycin. C2C12 cells were transfected with 100 nM concentration of miRNA mimics, miRNA inhibitor, NC or INC, which were synthesized from Gene Pharma (Shanghai, China) using Lipofectamine 2000 (Invitrogen, Carlsbad, CA, USA) in serum-free Opti-MEM. After 6 h of culture, use DMEM, 10% FBS and 1% penicillin and streptomycin to replace serum-free Opti-MEM.

miR-29a mimics forward, 5′- UAGCACCAUCUGAAAUCGGUUA -3′, and 

reverse, 5′- ACCGAUUUCAGAUGGUGCUAUU -3′; 

miR-29b mimics forward, 5′- UAGCACCAUUUGAAAUCAGUGUU-3′, and 

reverse, 5′-CACUGAUUUCAAAUGGUGCUAUU -3′;

negative control (NC) forward, 5′-UUCUCCGAACGUGUCACGUTT-3′ and 

reverse, 5′-ACGUGACACGUUCGGAGAATT-3′.

miR-29a inhibitor, 5′-UAACCGAUUUCAGAUGGUGCUA -3′,

miR-29b inhibitor, 5′-AACACUGAUUUCAAAUGGUGCUA -3′, 

inhibitor NC, 5′-CAGUACUUUUGUGUAGUACAA-3′.

### 2.7. Luciferase Reporter Assay

HEK293T cells were purchased from Beijing’s Cell Resource Center. HEK293T cells were cultured with DMEM, 10% FBS and 1% penicillin and streptomycin. HEK293T cells were transfected using the Lipofectamine 2000 reagent with psi-CHECK2^TM^ plasmid and miRNA mimics or miRNA inhibitor. Luciferase activity were measured by the Dual Luciferase Assay System (Promega, Madison, WI, USA). The data were normalized to the firefly luciferase signal.

### 2.8. Q-PCR 

Expression analysis of miRNA was based on Q-PCR (reverse transcription by stem-loop method). miR-29a RT stem-loop primer,

CTCAACTGGTGTCGTGGAGTCGGCAATTCAGTTGAGTAACCGAT; miR-29b RT stem-loop primer, CTCAACTGGTGTCGTGGAGTCGGCAATTCAGTTGAGAACACTGA; U6 RT primer, CGCTTCACGAATTTGCGTGTCAT). Q-PCR were conducted using a Roche LightCycler480 Real-Time PCR system. The primers sequences are as follows: 

miR-29a forward 5′-TCACGTAGCACCATCTGAA-3′

miR-29b forward 5′- TCACGTAGCACCATTTGAAA -3′

miR-29a/miR-29b reverse 5′-TCAACTGGTGTCGTGGAGT-3′; 

U6 forward 5′-CTCGCTTCGGCAGCACA -3′ 

U6 reverse 5′- AACGCTTCACGAATTTGCGT -3′. U6 was used as the internal standard.

The primers sequences of mRNA are as follows: 

Col4a1, 5′-TGGGAAAGACGGTGAAAAAG-3′ and 5′- AAAGGCATGGTGCCTATCAC-3′; 

Col4a2, 5′-CATCCGTCGGAGATGAAGAT-3′ and 5′- CAAACAGGAAGCCATCTGGT-3′; 

GAPDH, 5′-GGCTGCCCAGAACATCAT-3′ and 5′-CGGACACATTGGGGGTAG-3′. GAPDH was used as the internal standard.

### 2.9. Western Blotting

The following antibodies were used: anti-GAPDH (2118S, CST), and anti-Collagen IV (ab6586, abcam).

### 2.10. Statistical Analysis 

Data were analyzed using Student’s *t*-test with SPSS.

Calculating Cross Section Area (CSA) with Image J Software.

## 3. Results

### 3.1. Overexpression of the miR-29ab1 Cluster Leads to Skeletal Muscle Dysplasia

**Figure 1:**
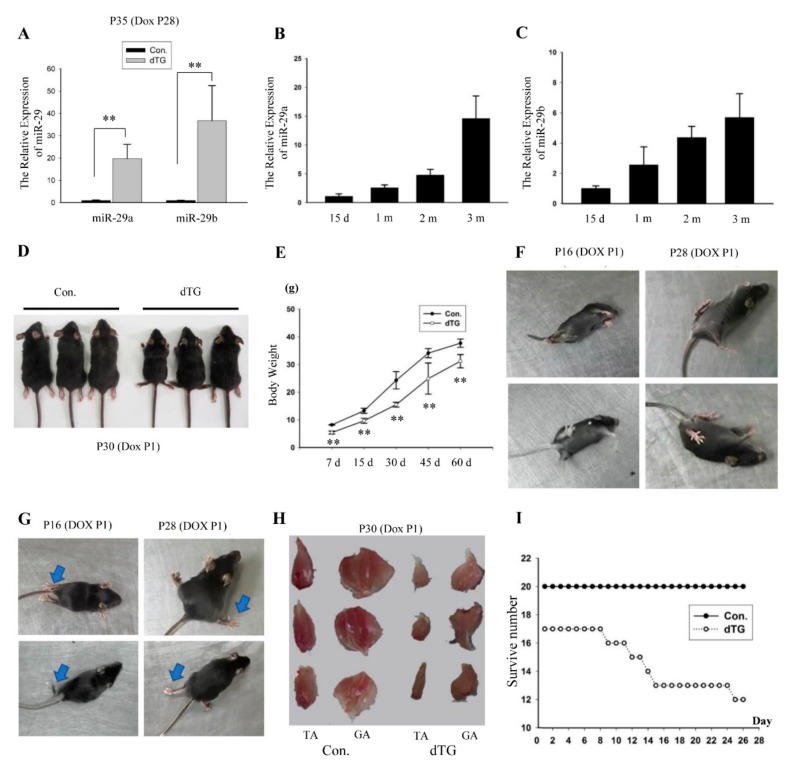
Overexpression of the miR-29a,b1 cluster leads to skeletal muscle dysplasia in juvenile mice. (**A**) Q-PCR analysis of miR-29a, b showing that miR-29a and miR-29b were induced in the tibialis anterior. The dual transgenic (dTG) mice were treated with Dox at P28 (28 day of postnatal development). The tibialis anterior were collected at P35. **, *p* < 0.01. The values represent the mean ± SEM (n = 3). (**B**,**C**) Measurement of miR-29a, b in the tibialis anterior muscles in mice at different ages. d, day(s); m, month(s). (**D**) Size comparison of 30-day-old control and dTG mice. (**E**) Body weight quantification over time; n = 10 for 7 days, n = 6 for other timepoints. **, *p* < 0.01. (**F**,**G**) Representative photograph of the dTG mice crawling and turning over. (**H**) Comparison of tibialis anterior muscles and gastrocnemius muscles between control and dTG mice. (**I**) Number of surviving control and dTG mice over time. n = 20 for control mice, n = 17 for dTG mice.

To develop a mouse model that can reproduce features of UCMD, we used the mice generated by Tet-on system in which the overexpression of the miR-29a, b could be induced in the dual transgenic (dTG) mice muscle fibers with temporal specificity (Figure 1A). The level of miR-29 was 30 times higher in the dTG mice than in the control.

The expression of miR-29a, b increased in developing postnatal muscles (Figure 1B,C) [19,20], then we induced miR-29ab1 cluster overexpression on postnatal first day. The Dox-treated dTG mice began to exhibit small size (Figure 1D), low body weight (Figure 1E), rolling over the body hardly (Figure 1F, Supplementary Video S1) and dyskinesia (Figure 1G, Supplementary Video S1). The muscle mass of the dTG mice was significantly reduced (Figure 1H). In total, 29% of the dTG mice did not survive one month (Figure 1I), and these mice displayed respiratory disturbances before they die (Supplementary Video S2).

**Figure 2:**
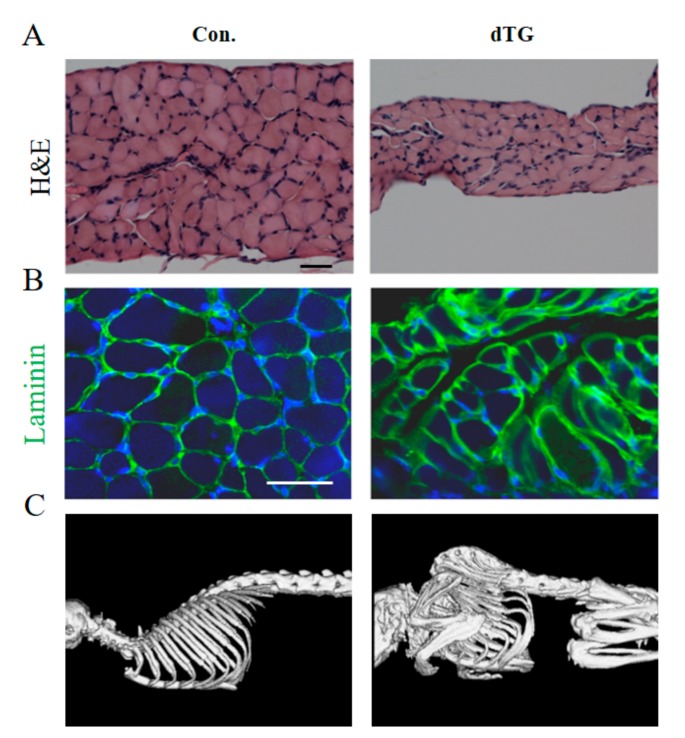
Diaphragm and skeleton of dTG mice display anomalies. (**A**) H&E staining of diaphragm muscles. Scale bars: 50 μm. (**B**) Immunofluorescence detection of Laminin expression in diaphragm muscles (green represents Laminin), scale bars: 50 μm. (**C**) X-ray photograph of the control and dTG mice.

Skeletal muscle related to breathing include the external intercostal muscles, internal intercostal muscles and diaphragm. The diaphragm is the main contributor to breathe [21]. The diaphragm in the dTG mice was significantly thinner than that of the controls, and the fibers were loosely arranged (Figure 2A). In addition, fibrosis was observed in the diaphragm of the dTG mice (Figure 2B).

The partially induced dTG mice displayed severe scoliosis and kyphos (Figure 2C). The Skeletal muscle were attached to the skeleton, indicating the abnormal development of skeletal muscle, which may lead to abnormal skeleton development [22].

### 3.2. Overexpression of the miR-29ab1 Cluster in Juvenile Mice Induces Skeletal Muscle Injury

The collagen VI-related myopathies include a range of diseases ranging from severe Ullrich muscular dystrophy to mild Bethlem myopathy [23]. Different miR-29 overexpression levels can mimic different disease states.

The level of miR-29 was 15–25 times higher in the dTG mice than in the control, and the dTG mice showed relatively moderate skeletal muscle damage. The dTG mice walked and turned over “normally”. When the “normal” dTG mice consumed physical energy by swimming, they turned over more slowly than the controls (Supplementary Video S3). Moderately damaged dTG mice can be considered a mouse model which can resemble pathology of Intermediate phenotype.

**Figure 3:**
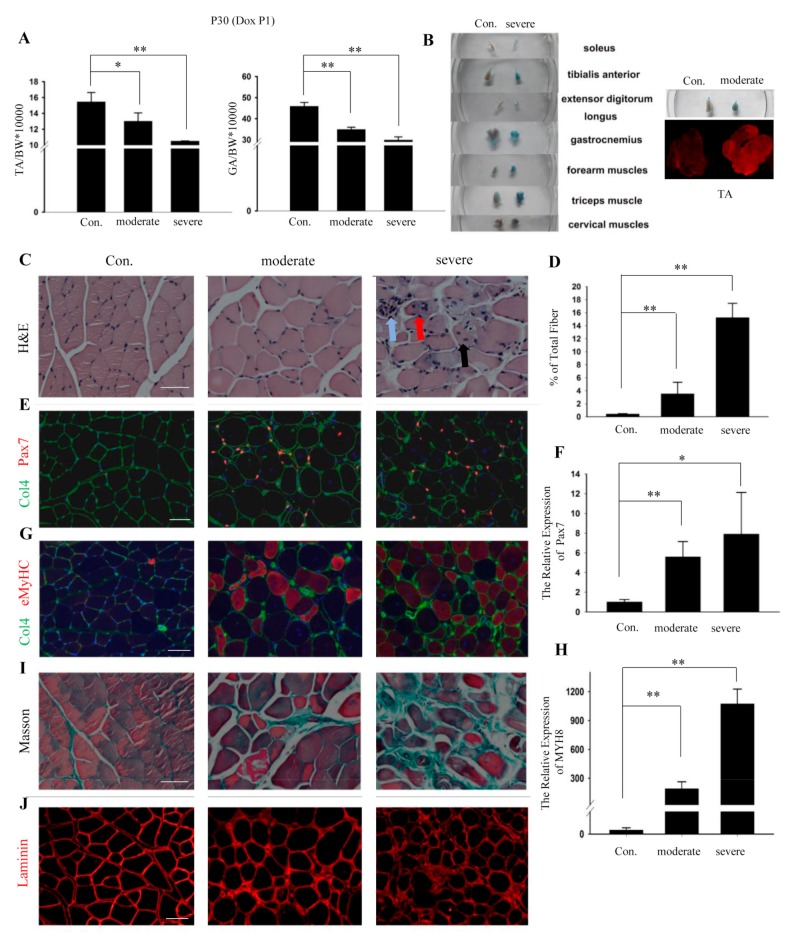
High miR-29 expression in juvenile mice induces skeletal muscle injury. (**A**) Quantification of tibialis anterior (TA) and gastrocnemius (GA) muscles weight at 30 days (n = 5). *, *p* < 0.05; **, *p* < 0.01. (**B**) Detection of myofibers damage in individual whole-body muscle following i.p. injection of 1% EBD 24 h prior to sampling. Comparison of tibialis anterior muscles between control and moderate dTG mice. And immunofluorescence analyses of EBD in the tibialis anterior muscles squash slide between the control and moderate mice. (**C**) H&E staining of tibialis anterior muscles. (**D**) Quantification of the rate for central nuclear myofibers. **, *p* < 0.01. (**E**) Immunofluorescence analyses of Pax7 (red) and collagen IV (green) in tibialis anterior muscles of control and dTG mice. Scale bars: 50 μm. (**F**) Expression analysis of Pax7. GAPDH was used as an internal normalized reference. The values represent the mean ± SEM (n = 3). *, *p* < 0.05; **, *p* < 0.01. (**G**) Immunofluorescence analyses of eMyHC (red) and collagen IV (green) in tibialis anterior muscles of control and dTG mice. Scale bars: 50 μm. (**H**) Expression analysis of MYH8. (**I**) Masson staining of the tibialis anterior muscles. Scale bars: 50 μm. (**J**) Immunofluorescence analyses of Laminin in tibialis anterior muscles of control and dTG mice. Scale bars: 50 μm. Con.: control mice, moderate: moderately damaged dTG, severe: severely damaged dTG.

To better illustrate the advantages of the dTG mice as a mouse model, the severely and moderately damaged dTG mice are both described. Consistent with the exercise capacity, the ratios of the TA and GA muscles in the severely and moderately damaged dTG mice were also stunted (Figure 3A).

To further illustrate the state of the skeletal muscle phenotype, Evans Blue Dye (EBD) was used. EBD has been widely used as a tracer to evaluate cellular membrane permeability in multiple models of skeletal muscle injury [24]. One day before the sample collection, EBD was injected i.p. into 28-day-old dTG mice and control mice [16]. The severely damaged dTG mice exhibited EBD in multiple muscles (Figure 3B). The moderately damaged dTG mice also exhibited EBD staining (Figure 3B). The dye was observed in all TA muscles in the moderately damaged dTG mice (Figure 3B), suggesting that the permeability of the cell membrane was flawed.

H&E staining showed many pathological phenotypes in the TA muscles of the dTG mice. The number of muscle fibers containing centrally located nuclei (black arrow) was significantly higher in the dTG mice than that in the controls (Figure 3C,D). In total, >3% of all muscle fibers exhibited centrally located nuclei, which is a hallmark indicator of myopathy [25]. 

Normal fibers have a pentagonal or hexagonal shape, but the dTG mice exhibited fibers that were circular or oval (black arrow) (Figure 3C). The dTG mice displayed a disorganized fiber distribution, necrotic fibers (blue arrow), and split fibers (red arrow) (Figure 3C).

The activation of satellite cells and synthesis of eMyHC indicate the presence of muscle injury and regeneration [26,27]. In this study, satellite cells activation (Figure 3E,F) and eMyHC expression (Figure 3G,H) were observed in the TA muscles of the dTG mice. In addition, the Masson staining and Laminin immunofluorescence showed excess fibrosis and fiber shape changes in the TA muscles (Figure 3I,J).

**Figure 4:**
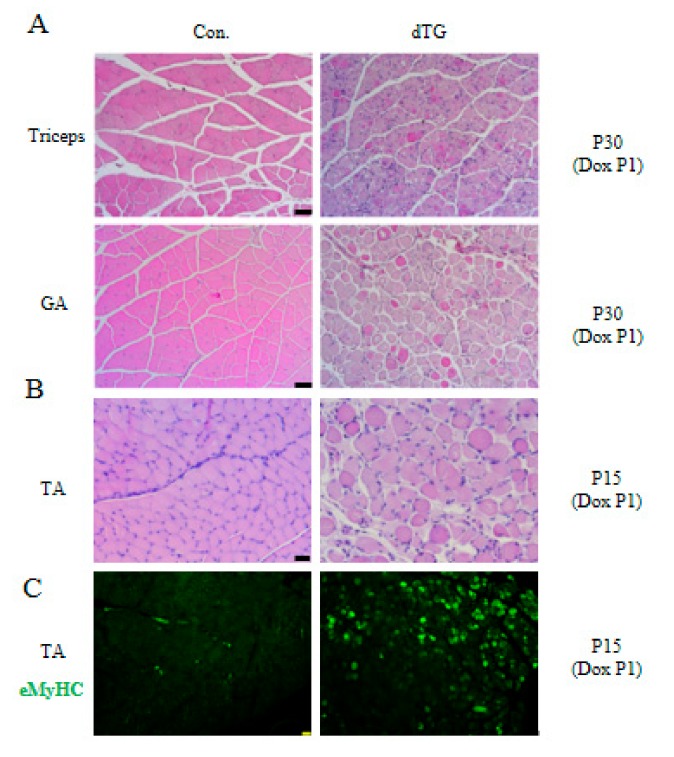
Multiple muscles of dTG mice display injuries. Scale bars: 50 μm. (**A**) H&E staining of Triceps and gastrocnemius (GA) muscles. (**B**) H&E staining of tibialis anterior (TA) muscles. (**C**) Immunofluorescence analyses of eMyHC (green) in tibialis anterior muscles of control and dTG mice.

We simultaneously examined the triceps of the forearm and gastrocnemius of the hindlimb, and H&E results showed serious muscle tissue injury in the induced dTG mice (Figure 4A). H&E staining and eMyHC immunofluorescence demonstrated severe skeletal muscle dysplasia and regeneration in the 15-day postnatal dTG mice (Figure 4B,C).

### 3.3. Col4a1 and Col4a2 Are the Direct Target Genes of miR-29

**Figure 5:**
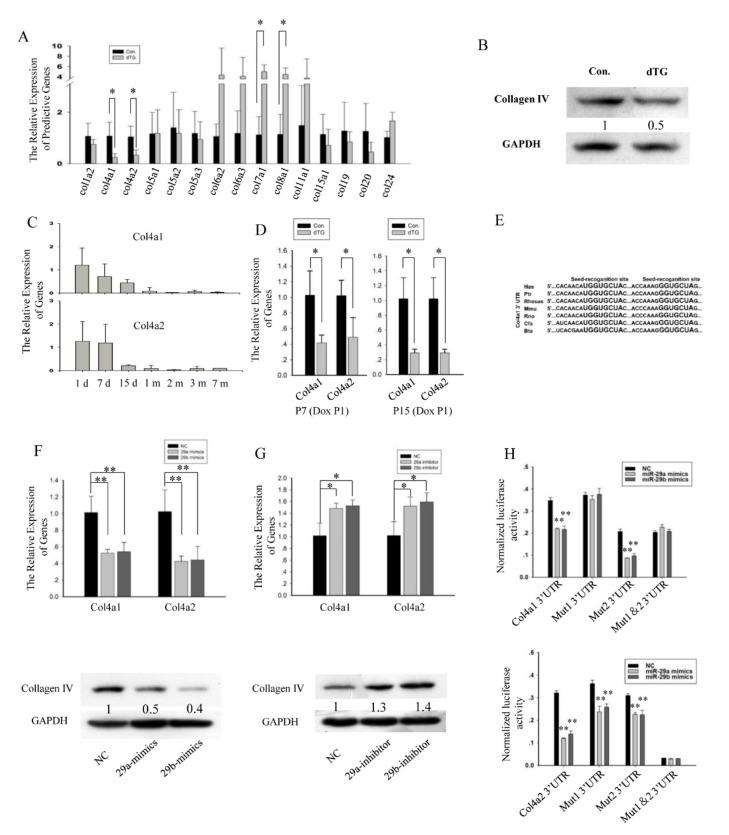
*Col4a1 and Col4a2* are the direct target genes of miR-29. (**A**) Expression analysis of miR-29 target genes related to collagen in the tibialis anterior muscles. The values represent the mean ± SEM (n = 3). *, *p* < 0.05. (**B**) Western blot showing the decrease in Col4 protein level in the dTG mice. GAPDH was used as an internal normalized reference. (**C**) The relative expression levels of *Col4a1* and *Col4a2* in the tibialis anterior muscles of mice at different ages were analyzed by Q-PCR. The values represent the mean ± SEM (n = 3). d, day(s); m, month(s). (**D**) Expression analysis of *Col4a1* and *Col4a2* in the tibialis anterior muscles. *, *p* < 0.05. (**E**) The binding sites in the *Col4a1* 3′UTR are indicated in bold, and all nucleotides in the binding sites are conserved across several species. Hsa, Homo sapiens; Ptr, Pan troglodytes; Rhesus, Macaca mulatta; Mmu, Mus musculus; Rno, Rattus norvegicus; Cfa, Canis lupus familiaris; Bta, Bos Taurus. (**F**) Expression analysis of genes related to protein degradation in the tibialis anterior muscles. *, *p* < 0.05; **, *p* < 0.01. (F) Expression analysis of *Col4a1* and *Col4a2* in C2C12 cells transfected with miR-29 mimics or negative control (NC). The values represent the mean ± SEM (n = 3). **, *p* < 0.01. (**G**) Expression analysis of *Col4a1* and *Col4a2* in C2C12 cells transfected with miR-29 inhibitor or INC (n = 3). *, *p* < 0.05. (**H**) miR-29 mimics influenced the relative luciferases activity of the *Col4a1*, mutant (Mut) *Col4a1*, *Col4a2*, and mutant *Col4a2* vectors. **, *p* < 0.01.

Subsequently, we determined the genes related to cellular membrane or extracellular matrix that were disturbed in the dTG mice. Prediction using TargetScan 6.2 revealed that the 3′UTRs of the mouse collagen genes contain conserved complementary sequences of miR-29. Thus, we measured the expression of these genes, and only the mRNA levels of *Col4a1* and *Col4a2* were significantly downregulated in the 1-month-old dTG mice (Figure 5A). The protein level of Col4 was also decreased in the dTG mice (Figure 5B).

Col4 is the main component of basement membrane [28], Col4 direct interact with collagen VI [29,30]. Heterozygous mutations in COL4 have been identified in both mouse models and families with porencephaly, angiopathy, nephropathy, aneurysms, and muscle cramps [31,32,33]. *Col4a1* and *Col4a2* were highly expressed in newborn mouse skeletal muscle (Figure 5C). In the dTG mice, the expression of *Col4a1* and *Col4a2* was decreased (Figure 5D), which destroyed the formation of the basement membrane.

The predicted binding sites of *Col4a1* (Figure 5E) and *Col4a2* are conserved in several species. Then, we investigated whether miR-29 directly inhibits *Col4a1* and *Col4a2*. According to the Q-PCR and Western blot analyses, miR-29 mimics inhibited the expression of *Col4a1* and *Col4a2* (Figure 5F), and the miR-29 inhibitor upregulated the expression of *Col4a1* and *Col4a2* (Figure 5G).

We chose the psiCHECK^TM^-2 vector to confirm the direct inhibition of 3′UTRs target site. The putative 3′UTRs target site or mutation site was cloned downstream of the reporter gene. Then, we co-transfected the psiCHECK^TM^-2 vector (wild-type *Col4a1* and *Col4a2* or mutant *Col4a1* and *Col4a2*) with miR-29 mimics or negative control (NC) into HEK293T cells.

Twenty-four hours after the transfection, the luciferase activity was measured. The relative luciferase activity in the cells that were co-transfected with the miR-29 mimics and wild-type *Col4a1* and *Col4a2* vector was significantly lower than that in the cells co-transfected with NC and wild-type *Col4a1* and *Col4a2* vector (Figure 5H). According to the relative luciferase activity, miR-29 inhibited *Col4a1* via the 33 bp-40 bp binding region at the 3’UTR, whereas the inhibition of *Col4a2* occurred through two binding regions at the 3’UTR, i.e., 43 bp-49 bp and 214 bp-220 bp.

## 4. Discussion

The collagens are essential for the extracellular matrix. UCMD is an early onset disease, which caused by loss of collagen VI. The collagen VI-related myopathies encompass a range of diseases ranging from severe UCMD to mild BM [23]. Disease models constructed by knocking out the *Col6a1*, *Col6a2*, or *Col6a3* gene, and none of these mice could mimic UCMD [8,9,10,11,12].

The Col6a1 mutant Landseer dogs can reproduce the human UCMD phenotype, and zebrafish model constructed by mutating Col6a1 can also mimic the human phenotype [34,35,36]. So far, no studies have been able to explain why Col6a1 mutations are so variable in different species [6]. Well, based on phenotype of *Col6a1*, *Col6a2*, and *Col6a3* mutant mice model and human UCMD, we could speculate that the function of collagen is not the same in human and mouse species. However, the specific mechanism needs to be further studied.

Although there are more severe animal models in dogs and zebrafish, the mouse disease model is irreplaceable for scientific research. Because of this, although the disease model mice cannot simulate human UCMD, the model mice still provide a major role in promoting the research and treatment of UCMD [37,38,39,40,41,42,43].

Collagen VI associates with the ECM around muscle cells and interacts with several other matrix constituents in human beings [29,30,44]. Efficient contraction of skeletal muscle is closely related to the muscle fibers, the surrounding ECM, and the coordination between them [45,46,47]. So, the essential cause of UCMD is the abnormality of extracellular matrix-related proteins.

The members of the miR-29 family are closely related to extracellular matrix-related proteins. Low expression of miR-29 can cause fibrosis in many tissues and organs fibrosis of various tissues and organs, including liver [48,49,50], lung [51,52,53], heart [54,55], kidney [56], skin [57,58], trabecular meshwork [59], and bone [60,61]. At the same time, the down-regulation of the expression of miR-29a and miR-29c in patients with primary skeletal muscle disease is also directly related to skeletal muscle fibrosis [62]. Therefore, we constructed a UCMD model by overexpressing miR-29 to inhibit extracellular matrix-related proteins. The dTG mice skeletal muscle uncovered a significant myopathic phenotype. More importantly, we can mimic the spectrum of the collagen VI-associated myopathy phenotype by adjusting the expression of miR-29.

Similar to UCMD patients, the miR-29ab1 cluster dTG mice exhibit dyskinesia, dyspnea, and kyphosis. Because of the abnormal development of respiratory-related skeletal muscle, the overexpression of miR-29 affected the life span of mice. However, the life span of the previous model mice was not affected, and there was also no significant abnormality in their walking ability [6]. Skeletal muscle affects the development of bone, many human being muscular dystrophy display abnormal development of bone, the phenotype of bone is caused by gene loss or myopathy that is in dispute [21,23,63,64,65]. The muscle-specific miR-29ab1 cluster dTG display the scoliosis and kyphos also mean myopathy can be significantly and enough interrupt the bone development. So, the miR-29ab1 cluster dTG mice simulate the human clinical and pathological characteristics of UCMD patients.

Analysis of multiple target genes that miR-29 may inhibit and we found that miR-29 did not inhibit collagen VI, but inhibited the expression of *Col4a1* and *Col4a2*. Col4 is a component of the basement membrane of various tissues and organs [66]. Col4 forms a microfibrillar network which interacts with other ECM components, including collagen VI [30,67].These different types of proteins can work together to support and protect the integrity of muscle. Col4 is a critical role in the ECM formation and cell arrangement in skeletal muscle. At the same time, the mutation of Col4a1 in mice can also cause skeletal muscle dysplasia [33,68], which further confirms our results. Therefore, a mouse model that resemble pathology of UCMD can be constructed by inhibiting the expression of *Col4a1* and *Col4a2* in the skeletal muscle.

In this study, the miR-29ab1 cluster dTG mice displayed a significant myopathy and downregulated collagen expression, suggesting that these muscle-specific miR-29ab1 cluster dTG mice constitute an excellent mouse model that can recapitulate features of UCMD. The dTG mice can provide suitable materials for the drug development of related diseases and provide reference for understanding the pathogenesis of human diseases.

## 5. Conclusions

Col4 is important for the postnatal skeletal muscle formation, the insufficient of *Col4a1* and *Col4a2* could cause some phenotypes of UCMD, and the miR-29ab1 cluster dTG mice were also demonstrated to be an excellent model that resemble the pathology of UCMD.

## Supplementary Materials

The Supplementary Materials are available online at https://www.mdpi.com/2073-4409/8/5/459/s1 (Supplementary Videos S1–S3).
